# Supplementary figures and images for: GSEA and the coexpression network approach identify novel pathway connections of molecular processes affected in Porto-sinusoidal vascular disease
Source: PLoS One. 2026 May 29;21(5):e0347338. doi: 10.1371/journal.pone.0347338 (PMC13220999; doi:10.1371/journal.pone.0347338)

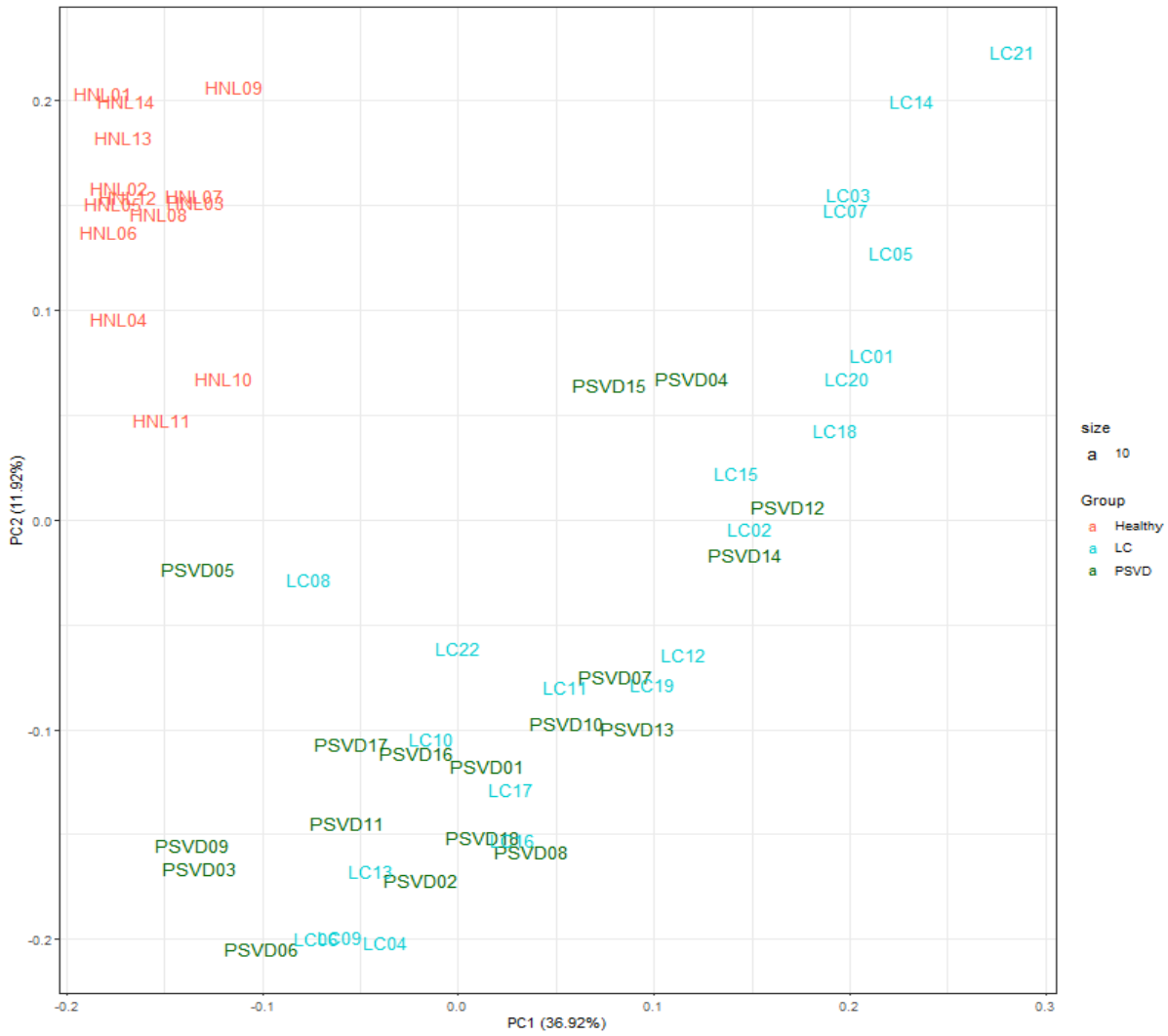

Supplement: S1 Fig — PCA was performed on normalized transformed gene expression data. The PCA plot above represents the variance explained by the top two components. (PDF) [file pone.0347338.s001.pdf]

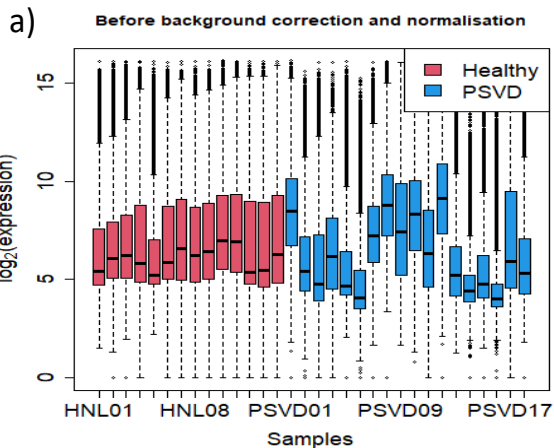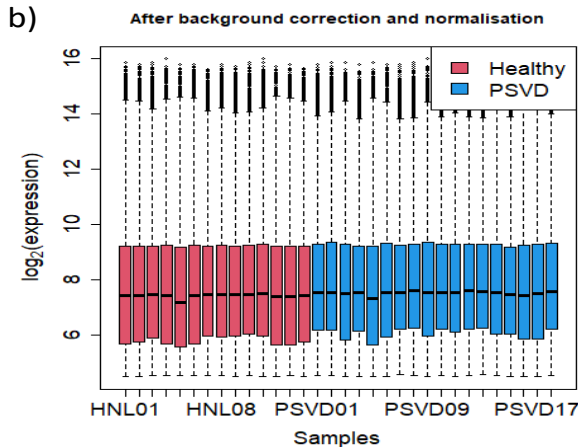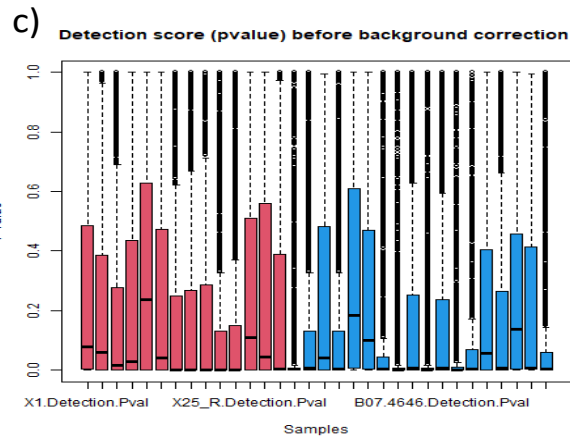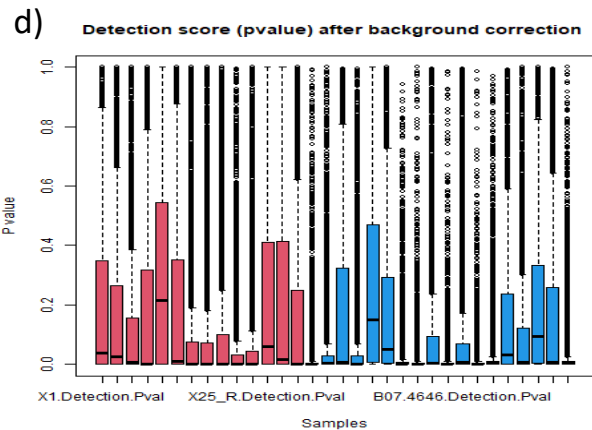

Supplement: S2 Fig — (A)Distribution of the gene expression data before and after normalization. X-axis represents the samples (pink – Healthy liver biopsies and blue- PSVD liver biopsies). Y-axis represents the genes expression values in logarithmic scale. (B) Distribution of the detection p-value before and after normalization. X-axis represents the samples (pink – Healthy liver biopsies and blue- PSVD liver biopsies). Y-axis represents the p-values of the probes used to measure the gene expression of the samples. (PDF) [file pone.0347338.s002.pdf]

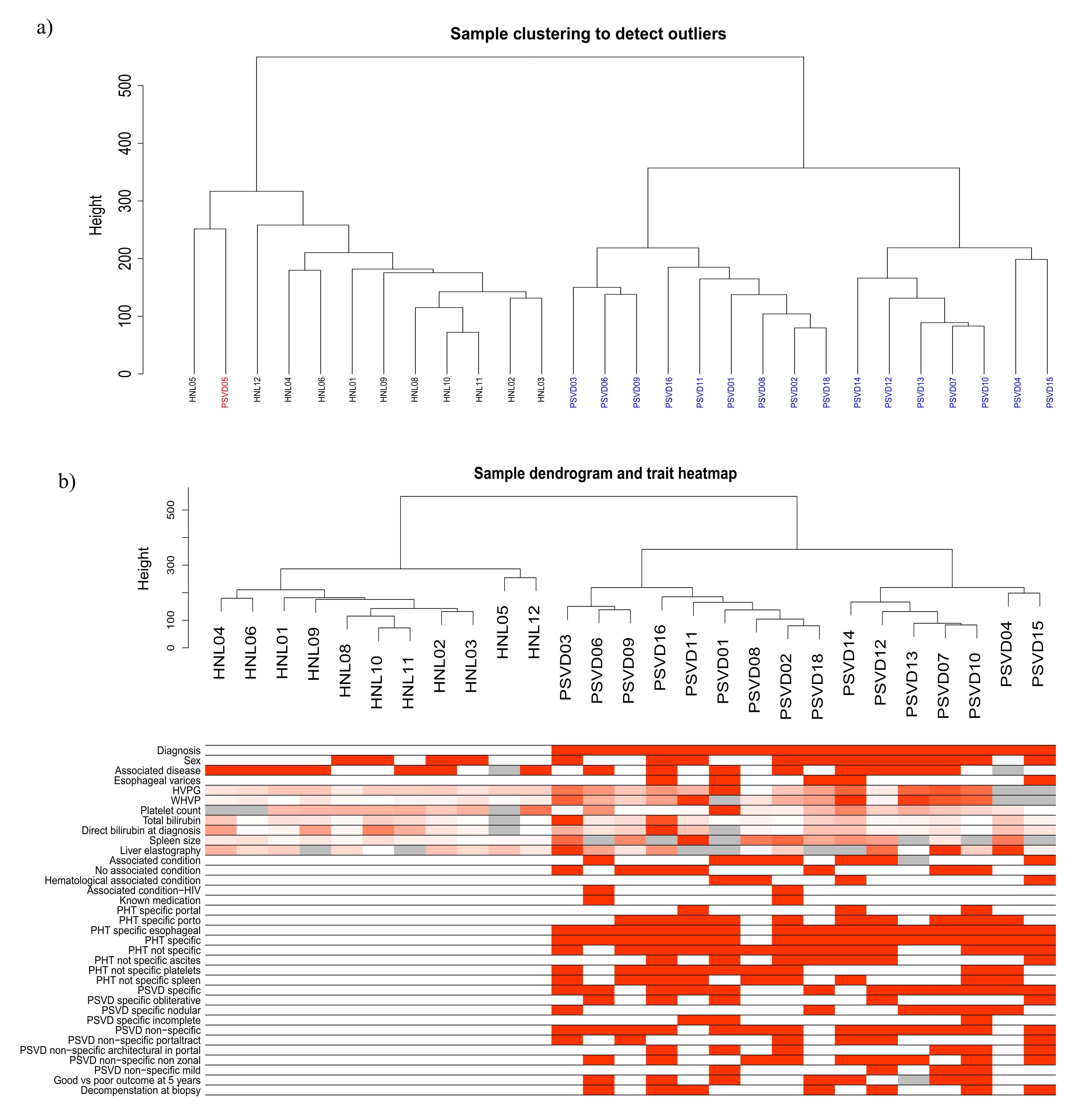

Supplement: S3 Fig — (a) Dendogram of the sample clustering. Sample PSVD05 (shown in red) was removed from the analysis given that it was clustering with the healthy liver samples. (b) Dendogram representing the sample clustering after outlier removal against the clinical variables visualized in the rows. (JPG) [file pone.0347338.s003.jpg]

Cluster Dendrogram

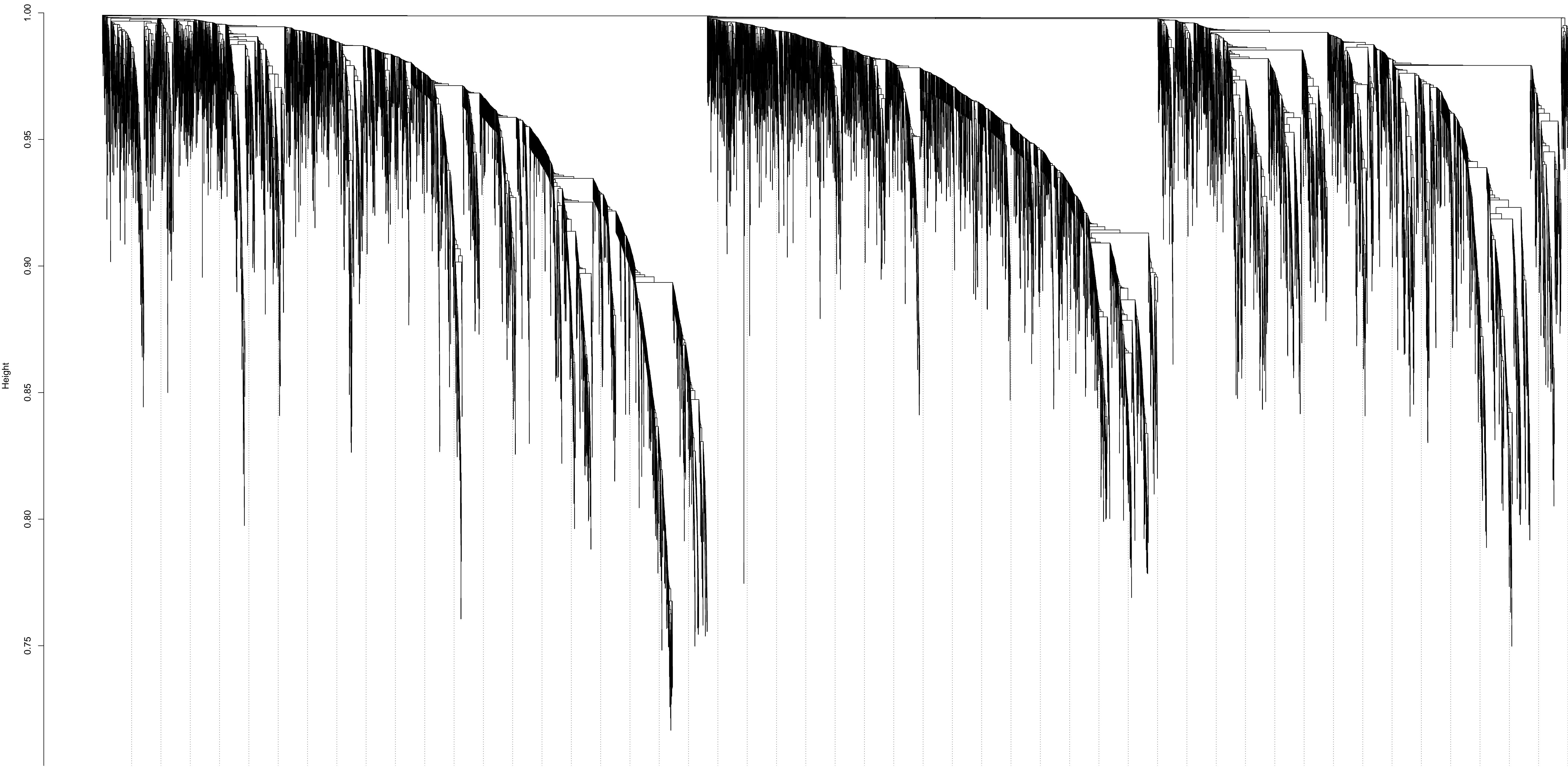

Dynamic Tree Cut

Merged dynamic

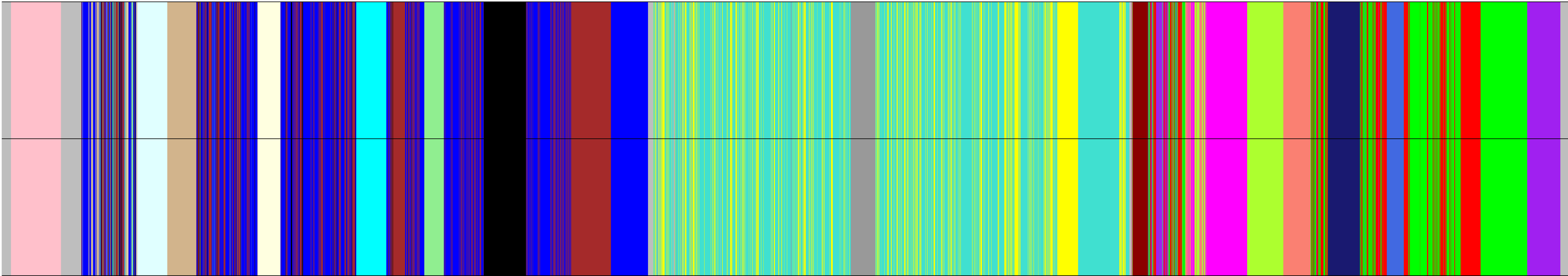

Supplement: S4 Fig — Hierarchical tree (average linkage) using dynamic tree cutting method was used for module detection. The Dynamic Tree Cut band represents the genes assigned to particular modules. 22 modules were detected. (PDF) [file pone.0347338.s004.pdf]

## Module–trait relationships

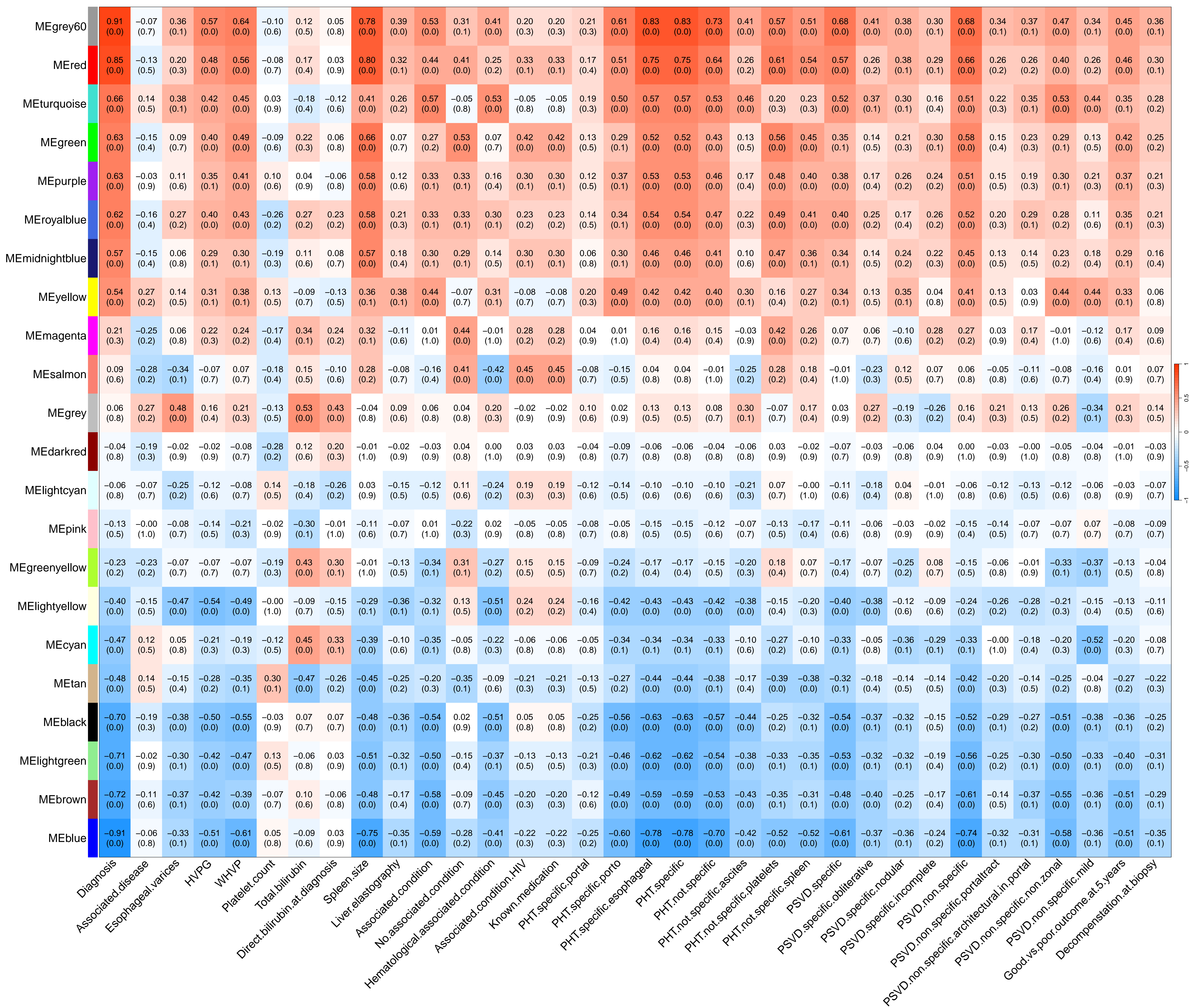

Supplement: S5 Fig — A heatmap of the module-trait relationship for modules significantly correlating to the diagnosis of PSVD on the y- axis and clinical variables on the x-axis. The color gradient on the heatmap represents the strength of the Pearson correlation coefficients. Number in each cell is the correlation and the p-value (in brackets). Hepatic venous pressure gradient (HVPG), wedged hepatic vein pressure (WHVP), portal hypertension (PHT), porto-sinusoidal vascular diseases (PSVD). (PDF) [file pone.0347338.s005.pdf]

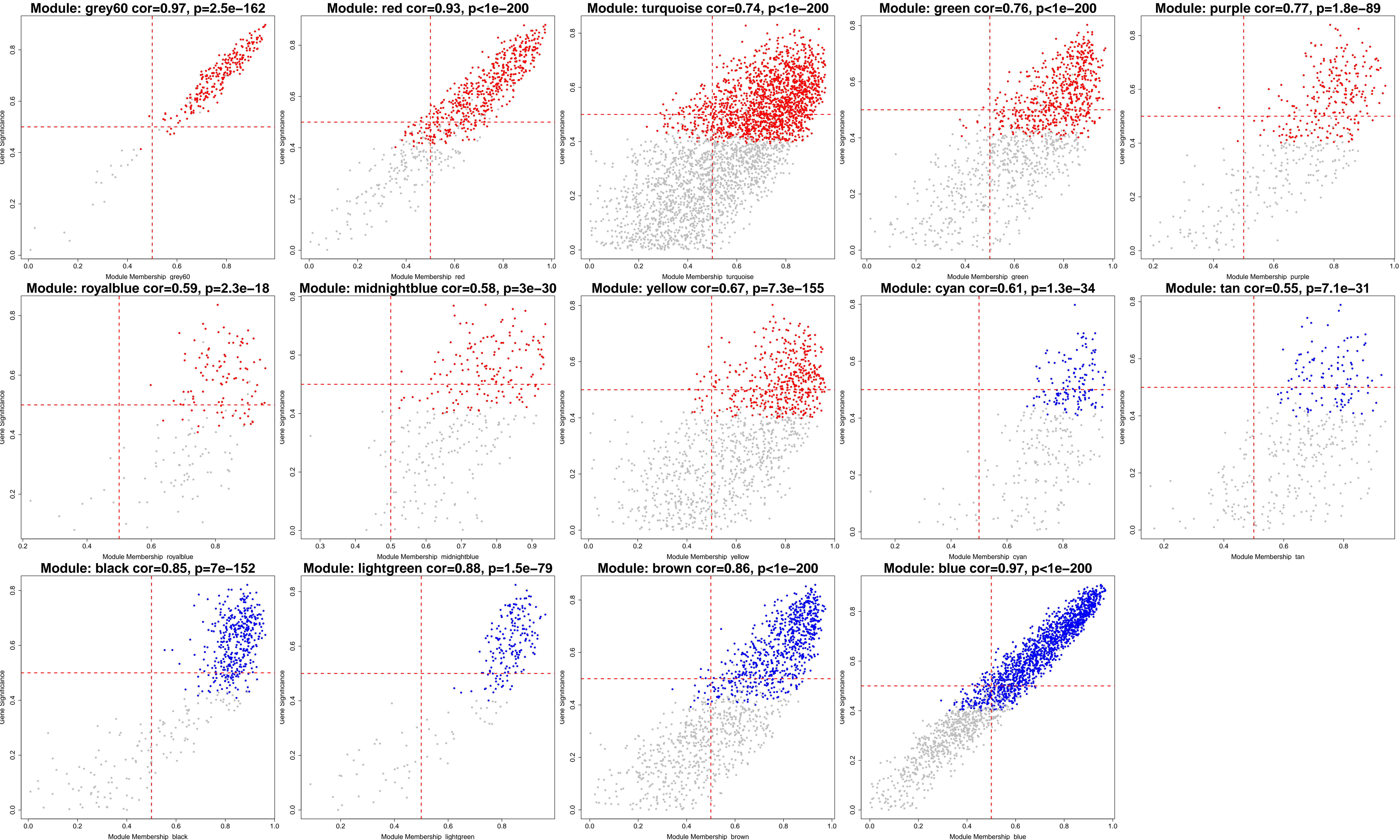

Supplement: S6 Fig — Each point represents a single gene, where GS reflects the correlation between gene expression and PSVD diagnosis, and MM represents the correlation between the gene and the module eigengene, indicating its connectivity within the module. Genes in the upper-right quadrant (GS > 0.5 and MM > 0.5) were designated as core (hub) genes, as they are both strongly associated with the trait and highly central within the module network.Data points are color-coded to reflect differential expression in PSVD: significantly up-regulated (red), down-regulated (blue) and non-significant (grey) genes. (PDF) [file pone.0347338.s006.pdf]
