# Supplementary material for: GSEA and the coexpression network approach identify novel pathway connections of molecular processes affected in Porto-sinusoidal vascular disease
Source: PLoS One. 2026 May 29;21(5):e0347338. doi: 10.1371/journal.pone.0347338 (PMC13220999; doi:10.1371/journal.pone.0347338)
Supplement: S7 Fig — Nodes represent core genes, colored by log-fold change (logFC). The red-bordered node is a previously reported gene, while large black-bordered nodes are the top five genes ranked by absolute(logFC) × node degree. Subclusters (circles) were identified using the Glay community detection algorithm, with functional enrichment and annotation performed via STRING.app. (PDF) [file pone.0347338.s007.pdf]

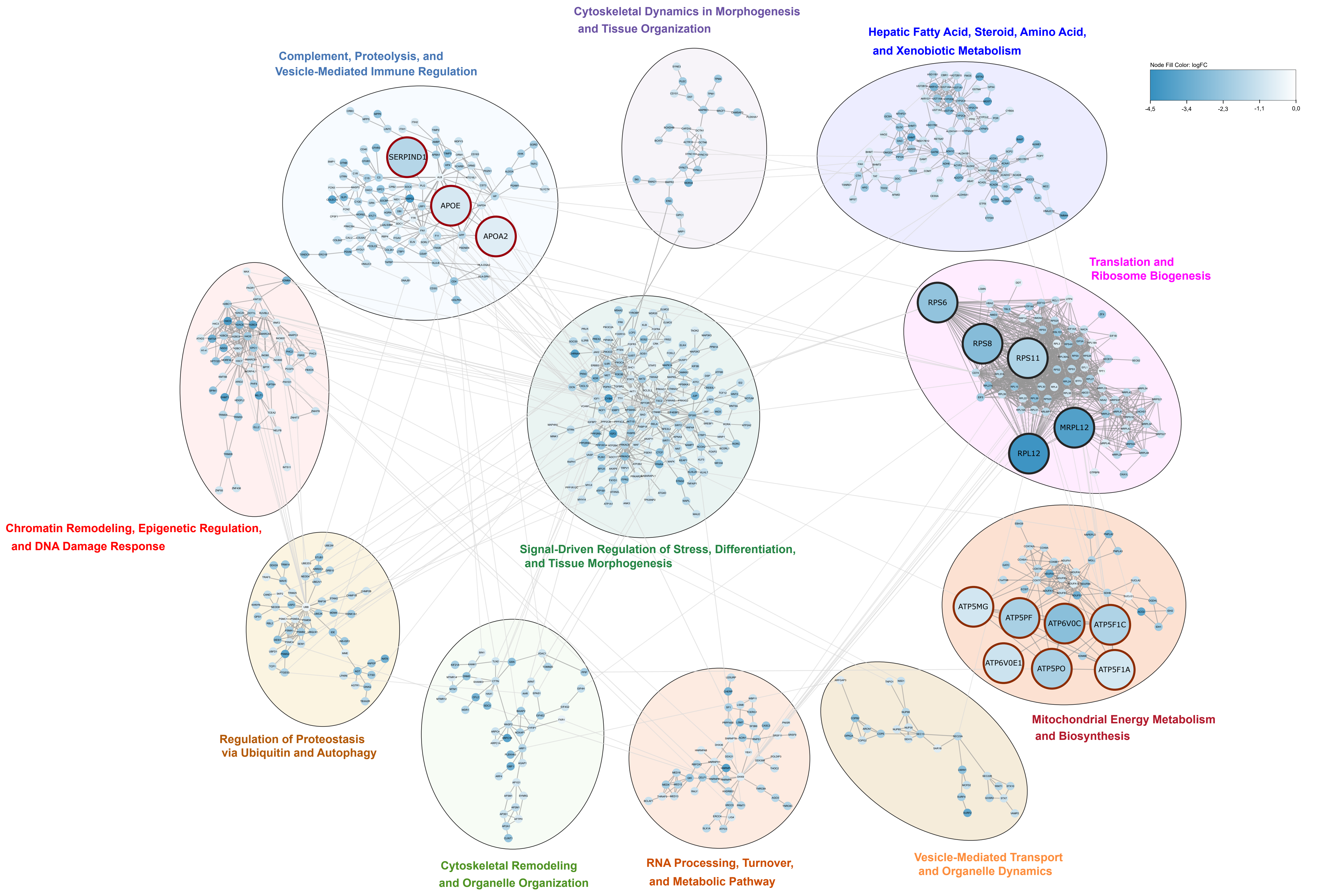
Cytoskeletal Dynamics in Morphogenesis
and Tissue Organization
Hepatic Fatty Acid, Steroid, Amino Acid,
and Xenobiotic Metabolism
Node Fill Color: logFC
-4,5
-3,4
-2,3
-1,1
0,0
Complement, Proteolysis, and
Vesicle-Mediated Immune Regulation
SERPIND1
APOE
APOA2
Translation and
Ribosome Biogenesis
RPS6
RPS8
RPS11
MRPL12
RPL12
Chromatin Remodeling, Epigenetic Regulation,
and DNA Damage Response
Signal-Driven Regulation of Stress, Differentiation,
and Tissue Morphogenesis
ATP5MG
ATP5PF
ATP6V0C
ATP5F1C
ATP6V0E1
ATP5PO
ATP5F1A
Mitochondrial Energy Metabolism
and Biosynthesis
Regulation of Proteostasis
via Ubiquitin and Autophagy
Cytoskeletal Remodeling
and Organelle Organization
RNA Processing, Turnover,
and Metabolic Pathway
Vesicle-Mediated Transport
and Organelle Dynamics
