# Supplementary material for: GSEA and the coexpression network approach identify novel pathway connections of molecular processes affected in Porto-sinusoidal vascular disease
Source: PLoS One. 2026 May 29;21(5):e0347338. doi: 10.1371/journal.pone.0347338 (PMC13220999; doi:10.1371/journal.pone.0347338)
Supplement: Attachment — Submitted filename: Response to Reviewers.pdf [file pone.0347338.s009.pdf]

We would like to thank the editor and reviewers for the detailed feedback on our manuscript! All changes in the manuscript are highlighted in red in the text.

Editor feedback – journal requirements

1. Please ensure that your manuscript meets PLOS ONE's style requirements, including those for file naming.

We will ensure that all files are named as required.

2. Please include your amended Funding Statement within your cover letter. We will change the online submission form on your behalf.

We added the amended funding statement in the cover letter, and we removed it from the acknowledgements.

3. We note that you have indicated that there are restrictions to data sharing for this study. PLOS only allows data to be available upon request if there are legal or ethical restrictions on sharing data publicly. We will update your Data Availability statement on your behalf to reflect the information you provide.

We would like to clarify that there are no data restrictions. Our study uses publicly available data from Hernández-Gea et al. (2021, DOI: 10.1016/j.jhep.2021.05.014), accessible through GEO under accession number GSE77627 (https://www.ncbi.nlm.nih.gov/geo/query/acc.cgi?acc=GSE77627).

We would appreciate it if our Data Availability statement can be updated accordingly.

4. We note that you have provided additional information within the Acknowledgements Section that is not currently declared in your Funding Statement. Please note that funding information should not appear in the Acknowledgments section or other areas of your manuscript. We will only publish funding information present in the Funding Statement section of the online submission form.

We added the amended funding statement in the cover letter, and we removed it from the acknowledgements.

5. Your ethics statement should only appear in the Methods section of your manuscript. If your ethics statement is written in any section besides the Methods, please delete it from any other section.

The ethics statement has been removed from the manuscript as we use the publicly available dataset from GEO:GSE77627 and the ethics statement is already published in their original publication.

6. We notice that your supplementary figures are included in the manuscript file. Please remove them and upload them with the file type 'Supporting Information'. Please ensure that each Supporting Information file has a legend listed in the manuscript after the references list.

We have made the appropriate changes and revised the supporting information in the required format as per journal guidelines.

7. We note that there is identifying data in the Supporting Information file < Supplementary File 1.csv>. Due to the inclusion of these potentially identifying data, we have removed this file from your file inventory.

The Supplementary File 1.csv contains the output from gene set enrichment analysis. This file does not contain any sensitive information.

Editor feedback - content

1. Reconsider the "weak" DEG category. Use standard FDR < 0.05 (or a justified threshold) for calling DEGs. If retaining "weak" DEGs, provide a strong biological or statistical rationale for the chosen logFC cutoffs and clearly differentiate them from significant DEGs in all analyses and visualizations. Avoid including them in functional enrichment summaries unless justified).

We thank the editor for this important comment. Upon reviewing our manuscript, we identified an error in how we described the DEG categorization. While we initially described "weak" DEGs as having adj.p-val > 0.05, all analyses were performed only on significant genes (adj.p-val < 0.05) with different log2FC thresholds.

To eliminate this confusion, we have removed the weak/strong categorization entirely. All analyses now use a single, standard threshold (|log2FC| > 0.58 & adj.p-val < 0.05) for defining DEGs. We have updated the entire manuscript and revised all figures (Figures 1, 2, and 4) to reflect this simplified approach, focusing only on significantly up-regulated, down-regulated, or unchanged genes.

2. : For genes without prior direct links to PSVD (e.g., GAGE12J, BARD1, RPS27A, GHRL, IL6, CD19, CDC6, TOP2A), rephrase conclusions. Focus on their statistical ranking and potential biological relevance based on known functions, but avoid labeling them as "novel" findings for PSVD without supporting evidence. Use phrases like "potential candidate", "statistically prioritized", or "suggestive of involvement".

We agree with the editor. We have, therefore, rephrased the conclusion section to include the potential candidate genes.

3. Ensure all figures are clear, well-labeled, and legends are unambiguous (especially Fig 2 volcano plot colors).

We have removed the DEG categories and thereby modified the volcano plot figure to now include only four categories significantly up-regulated, down-regulated, not significant, and not changed genes. The figure and legends should be clearer now.

4. Critically assess if all panels in Fig 5 are essential. Consider focusing on the core genes (5b).

We agree that splitting this information into two figures is not ideal. However, we do believe it is relevant to show the total number of modules' genes as well as the selected core genes. Therefore, we have combined both in one plot showing the total number of genes and DEGs for each module (stacked bar plot) and next to it a bar with only the core genes per module. This nicely demonstrates the effect of the core gene filtering.

5. Make absolutely certain that Figs 6 and 7 effectively communicate the complex correlation and PPI structures.

We have tried to improve the visual clarity of Figures 6 and 7 by optimizing network layout and styling to better display interconnections between clusters and reorganizing subclusters based on their connectivity patterns. These are standard PPI visualizations commonly used in literature, so we hope these modifications now effectively communicate the complex network structures to readers.

6. Elaborate on the GSEA vs. WGCNA immune pathway discrepancy. Discuss the potential biological and methodological reasons.

Yes, we agree and have included a separate section in the discussion to focus on GSEA vs WGCNA comparison, both biologically and methodologically. With a separate section for this discussion, we ensure that readers are able to better understand the complementarity of the approaches and the results and conclusions from our study.

7. Provide more nuanced biological interpretation of PPI subclusters beyond the functional labels. What specific interactions or potential regulatory roles do the hub genes play within their subclusters in the context of PSVD?

We appreciate the reviewer's suggestion. We believe the manuscript provides detailed functional characterization of PPI subclusters and discusses the roles of hub genes in the context of PSVD. Further speculation on specific protein interactions would be hypothetical without additional experimental validation, which is beyond the scope of this computational study.

8. If feasible, briefly discuss how specific module-clinical trait correlations (beyond just diagnosis) might inform the understanding of PSVD heterogeneity or severity.

-> We appreciate the reviewer's suggestion. However, we lack more detailed clinical data/information to discuss PSVD heterogeneity or severity and therefore cannot make any conclusions regarding this. Additionally, in our study we focus on PSVD diagnosis clinical trait, and we do observe the majority of clinical traits showing similar correlation to the key modules identified in our WGCNA analysis.

9. Sharpen the conclusion to emphasize the core finding of the immune/signaling vs. metabolic/translational/bioenergetic imbalance as the central pathogenic mechanism revealed by this integrated analysis, alongside the specific ribosomal and mitochondrial deficits. Position the "novel" genes as candidates for future validation.

Thank you for the suggestion. We rephrased the conclusion towards a clearer description of the core findings.

Reviewer feedback

Major Issues:
I don't have any major issues with the bioinformatics approach used by the authors. With that being said, the below comments should be addressed to improve the clarity of the manuscript.

• In GSEA, looks like the authors used both significant and weakly significant (p-value > 0.05) genes. What is the rationale behind using the weak significant candidates? Did the authors try using just the significant ones?

We thank the reviewer for this important comment. Upon reviewing our manuscript, we identified an error in how we described the DEG categorization. While we initially described "weak" DEGs as having adj.p-val > 0.05, all analyses were actually performed only on significant genes (adj.p-val < 0.05) with different log2FC thresholds.

To eliminate this confusion, we have removed the weak/strong categorization entirely. All analyses now use a single, standard threshold (|log2FC| > 0.58 & adj.p-val < 0.05) for defining DEGs. We have updated the entire manuscript and revised all figures (Figures 1, 2, and 4) to reflect this simplified approach, focusing only on significantly up-regulated, down-regulated, or unchanged genes.

• In the WGCNA analysis, it is not clear how many modules the authors were able to find. In Section 3.6 (Page 4, line 137), the authors specified finding 24 modules. However, in Section 4.3 (line 227), it's mentioned that 35 distinct modules were identified. Moreover, in the Supplementary Figure (Module-trait relationship heatmap) I could find only 22 modules. This needs to be clarified.

We thank the reviewer for identifying this mistake. We have corrected the manuscript to consistently report 22 distinct modules identified in our WGCNA analysis throughout all sections and supplementary materials.

• For constructing module PPI networks, the authors seem to have used different STRING confidence score thresholds. What is the reason/rationale for this?

We thank the reviewer for this question. The choice of STRING confidence thresholds was module-dependent, based on network size and connectivity. For the metabolic module, the high confidence *threshold (0.7) resulted in an excessively large network (for metabolic network; 0.7 string cut off – 1010 nodes and 4673 edges and with 0.9 cut of – 705 nodes and 2217 edges) that was difficult to visualize and interpret. We therefore applied a* very high confidence *threshold (0.9) for this module in the supplementary materials to focus on the most robust interactions. We have clarified this rationale in the Results section.*

• This manuscript can do with another table listing some of the top (relevant) enriched pathways (and individual genes from the DEGs/core genes) from both analyses. This can help identify robust pathways enriched in both methodologies.

We appreciate the reviewer's suggestion. However, given the substantial overlap in biological processes across enrichment terms (particularly in GO), a table format would be challenging to interpret clearly. Instead, we have restructured the Discussion section to better highlight the main biological processes consistently identified across both analytical approaches (WGCNA and GSEA analysis), emphasizing the robust pathways and mechanisms implicated in PSVD.

Minor comments:

• Looks like the placeholder texts form the journal manuscript template are still present, like Fig 1 caption in Page 6. This needs to be fixed.

This was due to the latex template; this is solved in the new version.

• In Section 3.3, looks like a negative sign is missing in describing the significantly weak downregulated genes. Also looks like the authors missed adding the word "weak" in this line (line 93). This should be corrected.

Thank you. We corrected this.

• Also, for the DEG categories, the authors used "significantly weak upregulated/downregulated" genes as the category names. Perhaps, using the phrase 'significantly weak' is confusing since the adjusted p-value thresholds used were not significant (> 0.05).

See comment above. In the analyses, those genes were actually significant, and we just overlooked the > vs. < typo. We apologize for this confusion.

• The sentence starting in line 228 (Section 4.3) seems to be left incomplete. This needs to be fixed.

Thank you for spotting this, we corrected the sentence.

• Formatting of Figure 5 legend seems to have been messed up in the manuscript.

We agree with the reviewer and based on the editor comments above to focus on the core genes from the WGCNA modules we have modified the bar plot figure to show the total and core genes together for all key modules.

• In the figure legend for Figure 6, the correlation values seem to have commas instead of decimal points.

Thank you for spotting this! We have corrected it.

• In figure S4 (cluster dendrogram), the label on the x-axis is cut.

We have edited the figure to make appropriate changes.

• Text on line 9 (Section 2) seems to have been scrambled. This needs to be fixed.

Thank you for spotting this! We have corrected this sentence.
