# Supplementary material for: GSEA and the coexpression network approach identify novel pathway connections of molecular processes affected in Porto-sinusoidal vascular disease
Source: PLoS One. 2026 May 29;21(5):e0347338. doi: 10.1371/journal.pone.0347338 (PMC13220999; doi:10.1371/journal.pone.0347338)
Supplement: Attachment — Submitted filename: Response to Reviewers_2.pdf [file pone.0347338.s010.pdf]

We would like to thank the editor and reviewers for the detailed feedback on our manuscript! All changes in the manuscript are highlighted in red in the text.

Editor feedback – journal requirements

Journal Requirements:

1. If the reviewer comments include a recommendation to cite specific previously published works, please review and evaluate these publications to determine whether they are relevant and should be cited. There is no requirement to cite these works unless the editor has indicated otherwise.

We will ensure to review the publication recommendations of the reviewer.

2. Please review your reference list to ensure that it is complete and correct. If you have cited papers that have been retracted, please include the rationale for doing so in the manuscript text, or remove these references and replace them with relevant current references. Any changes to the reference list should be mentioned in the rebuttal letter that accompanies your revised manuscript. If you need to cite a retracted article, indicate the article's retracted status in the References list and also include a citation and full reference for the retraction notice.

We will ensure to review the publication recommendations of the reviewer. One additional paper was added to the reference list.

First, the references were formatted to remove the [Journal Article] word from the bibliography section. Also, the doi for all references were checked and added in case missing.

Second,https://www.sciencedirect.com/science/article/pii/S0046817725002710?via%3Dihub- paper was added to reference list as we agreed with the reviewer in the section below that paper discussion would benefit from adding this paper to that section.

Additional Editor comments

1. Clarity on "Novel Pathway Connections": The title explicitly mentions "novel pathway connections," but the abstract doesn't clearly articulate what these novel connections are. While it mentions "coordinated dysregulation," it could be more explicit about the interrelationships found (e.g., the negative correlation between immune/signaling and metabolic modules).

We agree the editor with this comment and have according changed the Results and Conclusions section in the Abstract - This study revealed that PSVD is characterized by coordinated dysregulation, with an upregulated immune and signaling pathways activated alongside the concurrent suppression of metabolic, ribosomal, and mitochondrial programs.

2. In the "Results and Conclusion" section, explicitly state that the study identifies novel interconnections or antagonistic relationships between these pathway clusters. For example: "This study revealed a novel coordinated dysregulation in PSVD, characterized by the simultaneous activation of immune and signaling pathways alongside the suppression of metabolic, ribosomal, and mitochondrial programs, highlighting a critical antagonistic interplay between these systems."

We agree the editor with this comment and have according changed it in the Results and Conclusion section of the Abstract.

3. "Coordinated dysregulation" is a key finding. Can you highlight at how this coordination was identified (e.g., through module eigengene correlation)? phrase like: "Through module eigengene correlation network analysis, this study revealed a coordinated dysregulation..."

We agree the editor with this comment and have according changed it in the Results and Conclusion section of the Abstract. " In this study using both module eigengene correlation network analysis and GSEA, a novel coordinated dysregulation in PSVD was identified characterized by the simultaneous activation of immune and signaling pathways alongside the suppression of metabolic, ribosomal, and mitochondrial programs, highlighting a critical antagonistic interplay between these systems. "

4. While ribosomal proteins, ATP synthase, and serpin family members are mentioned, their significance could be further emphasized. Why are these particular molecules important?explain the consequences of their alteration. For example: "Alterations in ribosomal proteins, ATP synthase subunits, and serpin family members highlight translational, bioenergetic, and anticoagulant dysfunction, respectively, as core mechanisms."

We are not sure about this comment; it is the same sentence as the previous submission and was already changed according to the recommendations given. Could you please clarify?

5. After introducing Hernández-Gea et al. (lines 23-26), explicitly state the gap your study fills. For example: "While Hernández-Gea et al. provided crucial insights into vascular homeostasis and oxidative phosphorylation, a comprehensive, integrated understanding of the interplay between various dysregulated biological systems in PSVD, particularly the coordinated relationships between immune, metabolic, and signaling pathways, remains elusive. Our study aims to address this by..."

We agree the editor that further elaboration of our study aims after this sentence would benefit the clarity of the paper. We do not think it would be right to already conclude the relations between immune, metabolic and signaling pathways in PSVD, as we arrive to this conclusion only after we perform our analysis. We have rephrased the recommended addition to the following.

'"A recent study by Hernandéz-Gea et al., revealed previously unknown regulatory pathways affected in PSVD using co-expression analysis using gene expression data from healthy, PSVD and liver cirrhosis patients. The study indicated deregulation of pathways specific to vascular homeostasis and oxidative phosphorylation affecting the endothelial function. In this study, we aim to understand the connections between the dysregulated pathway/processes in specifically PSVD vs histologically healthy liver tissues using complementary approaches such

as GSEA and coexpression network analysis. Building upon these findings, our integrated approach would reveal a more complex, multi-systemic dysregulation, uncovering novel relationships, and refining the understanding of previously implicated pathways like oxidative phosphorylation."

6. Clarify the Relationship with Hernández-Gea et al. (Lines 23-26): This is the most direct comparator study. The introduction mentions its findings (vascular homeostasis, oxidative phosphorylation, endothelial function). Briefly mention how your approach differs or goes deeper. For example, did Hernández-Gea et al. use WGCNA but not GSEA, or did they focus on different aspects of network analysis? (The discussion section does address this, but a hint in the intro would be good).

We agree the editor with this comment and have according changed it in the Introduction section. Sentence mentioned above.

7. The discussion (lines 369-372, 394-396, 402-404) highlights differences and similarities with Hernández-Gea et al. (e.g., SERPINs, APOE/APOA2, ATP synthases). Consider weaving a very high-level preview of this into the introduction to further emphasize novelty. For instance, "Building upon these findings, our integrated approach reveals a more complex, multi-systemic dysregulation, uncovering novel antagonistic relationships between immune and metabolic processes, and refining the understanding of previously implicated pathways like oxidative phosphorylation." While true, the introduction could acknowledge the hypothesized contributors to PSVD's etiology, given the mention of associated conditions (lines 17-19). This would subtly connect to the pathways explored.

We agree the editor that the addition of the sentence would benefit the Introduction section in the context of the paper. We, however, modified the editors recommendtaion to not include the relations between immune and metabolic process yet in the context of PSVD a these were only identified in our analysis.

"Building upon these findings, our integrated approach would reveal a more complex, multi-systemic dysregulation, uncovering novel relationships, and refining the understanding of previously implicated pathways like oxidative phosphorylation."

8. Instead of just "The mechanism of disease development for PSVD is not known" (line 15), consider: "While the precise etiology of PSVD remains unknown, its development is strongly linked to vascular alterations within the liver and is frequently associated with disorders of immunity, blood diseases, prothrombotic conditions, and drug exposure [1], suggesting a multifactorial origin involving diverse biological processes." This naturally leads into why omics are needed.

We agree the editor with this comment and have accordingly changed it in the Introduction section.

"While the precise etiology of PSVD remains unknown, its development is strongly linked to vascular alterations within the liver and is frequently associated with disorders of immunity, blood diseases, prothrombotic conditions, and drug exposure [1], suggesting a multifactorial origin involving diverse biological processes."

9. Line 20: "Because PSVD incorporates a small, heterogeneous diseased patient group with varying physiological and histological features, there is sparse information regarding molecular pathways or processes affected in this condition." This is a slightly long sentence. Consider breaking it or rephrasing for better flow. "The small and heterogeneous nature of PSVD patient groups, coupled with varying physiological and histological features, has resulted in sparse information regarding the molecular pathways or processes affected in this condition."

We agree the editor with this comment and have accordingly changed it in the Introduction section.

"The small and heterogeneous nature of PSVD patient groups, coupled with varying physiological and histological features, has resulted in sparse information regarding the molecular pathways or processes affected in this condition. "

10. Ensure consistent use of "Porto-sinusoidal vascular disease" or its abbreviation "PSVD" throughout. The current usage is generally good.

We will ensure the consistent use of "Porto-sinusoidal vascular disease" or its abbreviation "PSVD" throughout the manuscript.

11. Line 23: "Hernand´ez-Gea et al." -> "Hernández-Gea et al." (check diacritics).

We thank the editor for identifying this mistake and have rectified the diacritics in the manuscript.

12. Line 28: "and next to link these genes to pathways thereby explaining the molecular mechanisms" -> "and subsequently linking these genes to pathways to explain the molecular mechanisms".

We agree the editor with this comment and have accordingly changed it in the Introduction section.

13. Line 31: "are coexpressed or change" -> "are coexpressed or exhibit coordinated changes".

We agree the editor with this comment and have accordingly changed it in the Introduction section.

Reviewer feedback

1. Reviewer #2: This is a well-designed and technically sound study; however, the Discussion would benefit from a focused revision to better contextualize the identified pathways within the evolving clinicopathologic understanding of porto-sinusoidal vascular disease (PSVD). In particular, the authors should more clearly link the GSEA- and coexpression network–derived pathways to established morphologic and clinical correlates of PSVD, including portal venopathy, sinusoidal remodeling, and altered hepatic microcirculation. Briefly addressing how these molecular signatures may overlap with or diverge from PSVD-like changes observed in other settings (e.g., post-transplant portal vasculopathy) would strengthen the translational relevance. The Discussion should also clarify whether the identified pathways support a primary vascular injury model, immune-mediated mechanisms, or secondary remodeling processes. Incorporation of recent clinicopathologic observations would enhance the manuscript, including: Khandakar B, Joldoshova A, Zhang X. Porto-sinusoidal vascular disease-like alterations in transplant liver biopsies: A clinicopathologic observation of transplant-associated portal vasculopathy. Hum Pathol. 2026;167:105984. doi:10.1016/j.humpath.2025.105984.

We appreciate the reviewer's suggestion. However, given the lack of the data related to portal venopathy, sinusoidal remodeling and altered hepatic microcirculation in our study, it would be difficult to make concrete conclusions on how the pathway/processes dysregulated would connect to these clinical traits. But we do agree that adding this is a future direction as part of the Discussion section for improving PSVD-related understanding of these clinical correlates would be essential for the disease prognosis, diagnosis and treatment options.

"While the molecular pathways identified in this study, including those derived from GSEA and coexpression network analyses offer insight into the dysregulated biological processes underlying PSVD, directly linking these signatures to established morphologic correlates such as portal venopathy, sinusoidal remodeling, and altered hepatic microcirculation remains challenging in the absence of corresponding histopathologic and hemodynamic data. Whether the identified pathways reflect a primary vascular injury, immune-mediated mechanisms, or secondary remodeling processes is an important question that the current dataset cannot definitively resolve. Notably, recent clinicopathologic observations have begun to characterize PSVD-like alterations in distinct settings, such as transplant-associated portal vasculopathy, highlighting the broader relevance of vascular remodeling signatures across hepatic disease contexts [51]. Future studies integrating transcriptomic findings with detailed histopathologic profiling and clinical vascular phenotyping will be essential to establish how these molecular signatures map onto the clinicopathologic spectrum of PSVD, and to clarify their potential utility in disease diagnosis, prognosis, and the identification of therapeutic targets. "
